# Digital Signal Compensation and Sounding Depth Analysis of Portable Frequency-Domain Electromagnetic Exploration System

**DOI:** 10.3390/s24020566

**Published:** 2024-01-16

**Authors:** Huipeng Liu, Jianxin Liu, Fang Wang, Leiyun Qian, Rong Liu

**Affiliations:** 1 School of Geosciences and Info-Physics, Central South University, Changsha 410083, China; liuhuipeng@zskk1953.com (H.L.); ljx@csu.edu.cn (J.L.); liurongkaoyan@csu.edu.cn (R.L.); 2 Yunnan Key Laboratory of Geotechnical Engineering and Geohazards, Kunming 650051, China; qianleiyun1@zskk1953.com; 3 Kunming Prospecting Design Institute of China Nonferrous Metal Industry Co., Ltd., Kunming 650051, China

**Keywords:** frequency-domain electromagnetic method, digital signal compensation, sounding depth

## Abstract

Low-resistivity objects produce eddy currents when excited with electromagnetic waves of a certain frequency and then generate an eddy electromagnetic field. A portable frequency-domain electromagnetic exploration system can be used to identify this eddy electromagnetic field, and then the low-resistivity objects can be positioned. At present, portable frequency-domain electromagnetic method (FEM) exploration systems use analog signal compensation, and the sounding depth is generally calculated using empirical formulas. In order to improve the rationality of signal compensation, this paper puts forward a digital signal compensation technology, including a device design, an information extraction method, and a primary field calibration method, and makes an exploration prototype based on the digital signal compensation technology. Using 10 nV as the minimum potential detection capability, the sounding depth of the portable FEM was analyzed, and it was found that when investigating a target with the same depth, a lower frequency required a larger emission current. If this could not be met, the sounding depth became smaller, and a phenomenon appeared in which the lower the operating frequency, the smaller the sounding depth. Through the detection of known underground garages, the apparent conductivity and normalized secondary field anomalies with higher sensitivity were obtained, which indicates that the detection system based on the digital signal compensation technology is effective in practical exploration. Via long-distance detection experiments on cars, it was confirmed that the sounding depth of the portable multi-frequency FEM in practical work indeed decreases with a decrease in the operating frequency.

## 1. Introduction

The frequency-domain electromagnetic method (FEM) is a method to identify low-resistivity objects or geological bodies by measuring eddy current electromagnetic waves based on the eddy current effect. A portable FEM detection system can be carried by the surveyor on their side, enabling rapid exploration to a depth of several meters below the surface, greatly improving the detection efficiency. At present, portable FEM detection systems include the EM series by Geonics in Canada [1,2,3], the GEM series by Geophex in the United States [4,5,6], Geovizer in Russia, and MIL-D in Italy. They have been widely used in urban underground metal pipelines [7] and metal structure exploration [8], soil pollutant distribution surveys [9,10], and special soil layer thickness [11] and ice layer thickness detection [12,13,14].

Early portable FEM exploration systems used analog signal compensation to extract normalized secondary fields, using electronic modules such as phase shifters, amplifiers, and phase-sensitive detectors to output in-phase and 90°-phase-difference amplitude signals at the same frequency as the reference signal [15,16]. Won et al. proposed to invert the reference coil and the receiving coil and then use a high-precision analog-to-digital converter to obtain the secondary and primary fields [4,17]. This compensation method is also analog signal compensation in essence. Although analog signal compensation can reduce the calculation pressure of embedded systems, it is difficult to make compensation coils accurately and calibrate accurate systems, resulting in insufficient detection accuracy. When strong electromagnetic noise is encountered in cities, weak secondary field signals are easily covered by the noise and cannot be effectively extracted [18].

Most scholars discuss the sounding depth of a portable FEM based on the generalized skin depth. Huang’s research holds that the detection depth of a portable frequency-domain FEM is proportional to the square of the skin depth [19]. In his description of the detection principle of a portable frequency-domain electromagnetic method, Li calculated the sounding depth via the skin depth. Based on this knowledge, Li used finite element simulation software to establish an infinite extension model with an upper layer of a 100 Ω·m thickness of 20 m and a lower layer of 20,000 Ω·m and calculated that a detection depth of 15 kHz could reach 41 m [20]. In actual use, the effective sounding depth of a portable frequency-domain electromagnetic detection system is generally less than 5 m. The above discussion of the exploration depth is based on the discussion of a normalized secondary field and does not take into account the reception ability of the portable FEM exploration system to the actual eddy current secondary field, so the application effect is not good.

In the past 10 years, Zhang has studied the key technologies of a towed frequency-domain electromagnetic method recording system [21], Qu et al. have studied a frequency-domain electromagnetic detection system used for the detection of metal-buried objects [22], and Chai has studied a frequency-domain electromagnetic detection system used for the detection of ice thickness [23]. Li studied the involvement and application of a wide-band frequency-domain electromagnetic detection instrument system [20]. All the above scholars produced detection prototypes in their research work, but they did not conduct in-depth research on signal compensation and sounding depth. In view of the lack of portable FEM analog signal compensation and the difficulty in determining the sounding depth, this paper proposes a digital signal compensation technology and presents a prototype. Based on the sensor of the prototype for receiving the secondary field signal, the exploration depth is analyzed. This is of great significance to enhance the practicability of portable FEM exploration systems.

## 2. How Portable FEM Works

**Figure 1:**
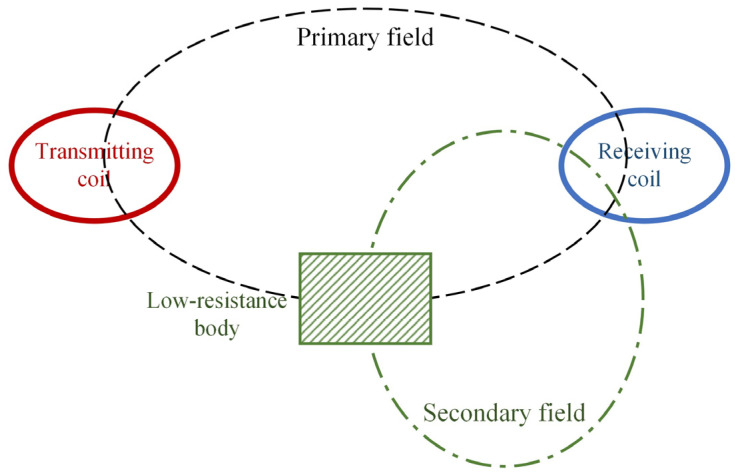
Principle of probing geologic bodies.

The working principle of the portable frequency-domain electromagnetic method is that a current with certain frequency information is supplied to the transmitting coil to generate an alternating magnetic field (primary field) around it, and the low-resistivity geologic body (referred to as the “low-resistance body”) forms eddy currents under the action of this primary field and generates a magnetic field (secondary field). As shown in Figure 1, the receiving coil is used to receive the primary field and the secondary field. When the distance between the transmitting coil and the receiving coil is unchanged, the primary field is unchanged. Therefore, the low-resistance body can be identified by analyzing the change in the secondary field on the profile.

The magnitude of the secondary field is not only related to the resistivity and buried depth of the geologic body but also causes the change in the secondary field when the primary field changes. In order to analyze the change in the secondary field, the normalized secondary field (the ratio of the secondary field to the primary field) parameter is usually used for the analysis. The normalized secondary field is commonly represented by the *PPM* symbol [24]:

(1) $$PPM=\frac{H_{S}}{H_{P}}=I+Qi$$

where $H_{S}$ is the secondary field, and $H_{P}$ is the primary field. After the fast Fourier transform of the time series data, the obtained frequency-domain data are complex, so $H_{S}$ and $H_{P}$ are complex, and the calculated *PPM* is also complex. I is the real component of the *PPM*, and Q is the virtual component of the *PPM*. Under the condition of uniform half-space, the calculation of the conductivity (σ) of the horizontal coplanar device can be simplified as [24]

(2) $$\sigma =\frac{4}{\omega \mu_{0}r^{2}}\;[PPM]$$

where ω is the angular frequency, $\mu_{0}=4\pi \times 10^{-7}$ H/m, and r is the transmit–receive distance.

The signal in the receiving coil includes a primary field and a secondary field, and the measurement of the primary field is the premise of extracting the secondary field. In order to obtain the primary field, a reference coil is added between the transmitting coil and the receiving coil, so that the transmit–receive distance, receiving area, and number of turns of the two coils meet the requirements [17]:

(3) $$\frac{A_{1}n_{1}}{X_{1}^{3}}=\frac{A_{2}n_{2}}{X_{2}^{3}}$$

where A, n, and X are the area, turns of the two coils, and transmit–receive distance of the reference coil and the receiving coil, respectively. The reference coil is close to the transmitting coil, and the received secondary field is much lower than the primary field. When the secondary field in the reference coil is approximately ignored and Formula (3) is satisfied, it can be considered that the amplitude of the primary field in the reference coil and the receiving coil is equal. The use of the reference coil to extract the information of the secondary field is called system compensation, and the reference coil is also called the “compensation coil”.

**Figure 2:**
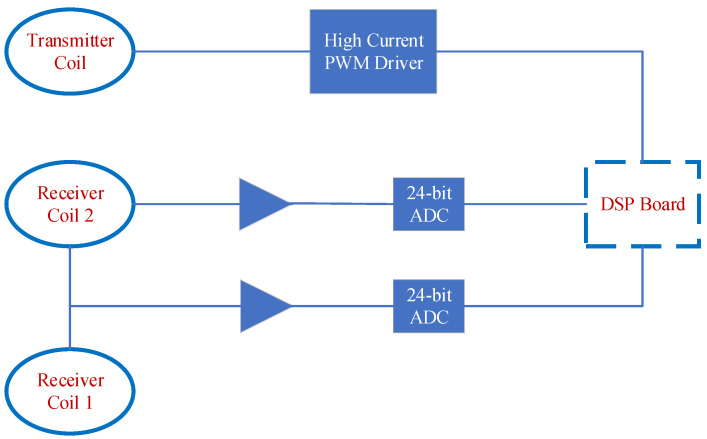
The working principle of analog signal compensation for GEM-2 device.

Portable FEM devices mostly use analog signal compensation methods: as shown in Figure 2, the phase shifter is used to adjust the phase of the reference coil, a phase-sensitive detector is used to screen the specified frequency in the receiving coil, and then an adjustable amplifier is used to calibrate the value, thereby obtaining the magnitude of the normalized real (in-phase) and virtual components (90° difference) of the secondary field.

In addition to the above analog signal compensation, Won proposed to reverse series the signal of the reference coil and the receiving coil so as to eliminate the primary field in the receiving coil (Figure 2). By receiving the signal after the primary field is eliminated and the signal from the reference coil, the normalized secondary field can be calculated [4]. This signal compensation method also belongs to analog signal compensation. The advantages are that the signal compensation structure is simple, and the operating frequency can be arbitrarily selected below the resonant frequency of the receiving coil, which is conducive to the realization of multi-frequency detection. However, the phase of the signal received by the reference coil and the receiving coil is inconsistent, and this compensation method produces obvious signal residue and cannot take advantage of the resonance frequency advantage of the receiving coil to enhance the receiving ability.

**Figure 3:**
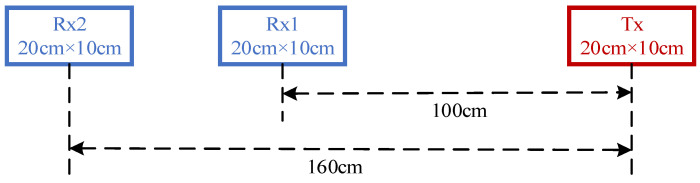
Design of simulated GEM-2 device.

The analog signal compensation method mainly has the following defects:

1. The secondary field in the reference coil is ignored. In order to reduce the mutual inductance between the reference coil and the transmitting coil, the reference coil also needs to be far away from the transmitting coil, and the number of turns of the coil is generally approximately 1/3 of the receiving coil. Ignoring the secondary field leads to a smaller normalized secondary field. Although the value of the normalized secondary field can be improved via system calibration, it cannot compensate for the defect of the reduced sensitivity of the secondary field.
2. Due to the space limitation of portable FEM detection equipment, the use of square coils is more conducive to increasing the area. However, there is a certain error in the design for the receiving of the square coil using Formula (3). In order to verify the size of this error, the square coil is designed by simulating the GEM-2 device, as shown in Figure 3. The turns ratio of the coil Rx1 and Rx2 actually calculated with the device according to Figure 3 is 0.354, while the turns ratio calculated according to Formula (3) is 0.244. If the Rx2 coil is 1000 turns, then the number of turns of the reference coil calculated according to Formula (3) is 110 turns less than the actual demand. This error results in the calculation of the secondary field containing the primary field information.

According to tests by Won, the GEM-2 device can suppress the primary field by up to 99% [17]. However, the secondary field signal caused by geological bodies is very weak and is different by more than two orders of magnitude from the primary field, and there is still a large number of primary field components in the remaining 1%. Therefore, analog signal compensation based on Formula (3) and ignoring the secondary field of the reference coil lead to insufficient accuracy.

## 3. Digital Signal Compensation

The digital signal compensation described in this paper consists of converting the analog signal of the reference coil and the receiving coil into a digital signal by using a high-precision analog-to-digital conversion device (ADC). The conversion coefficient of the primary field is obtained by calibrating the sensor. According to the measured induced electromotive force of the reference coil, the magnitude of the primary field signal in the receiving coil can be calculated by using the conversion coefficient, and the signal compensation can be realized. The realization of the digital signal compensation technology includes the device design, information extraction method, and primary field calibration.

### 3.1. Device Design and Information Extraction Method

**Figure 4:**
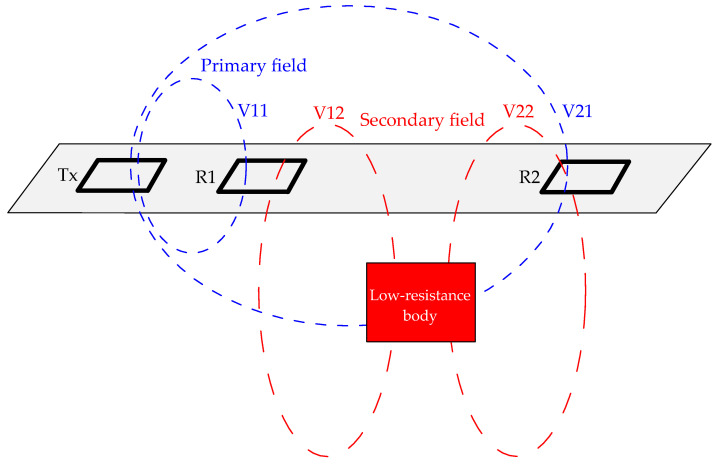
The working principle of the device.

The digital signal compensation receives the signals of the reference coil and the receiving coil. In order to improve the acquisition accuracy of the primary field signal, the reference coil is moved near the transmitting coil to more accurately obtain the primary field. Considering the difference in frequency response caused by different turns of the coil in multi-frequency operation, the number of turns, size, and material of the reference coil and the receiving coil are exactly the same. The working principle of the device is shown in Figure 4.

In Figure 4, Tx is the transmitting coil, R1 is the reference coil, and R2 is the receiving coil. Suppose that the induced electromotive force (EMF) in R1 is V1, including the primary field V11 and the secondary field V12, that is, V1 = V11 + V12. Suppose that the EMF in R2 is V2, including the primary field V21 and the secondary field V22, that is, V2 = V21 + V22. Suppose that the primary field in V2, amplified by *n* times, is the same as the primary field in V1, that is, V11 = *n*V21. After *n* times magnification, V2 minus V1, then,

(4) nV2−V1=nV22-V12

When the low-resistivity geological body is between the reference coil and the receiving coil, the secondary field in the reference coil is considered to be approximately equal to the secondary field in the receiving coil. Then, $\mathrm{nV}22-V12\approx \left(n-1\right)V22$. According to Formula (4), V22 and V21 can be calculated as

(5) $$V22=\frac{nV2\;-\;V1}{n-\;1}$$

(6) $$V21=V2-V22=V2-\frac{nV2-V1}{n-1}$$

By reading the EMF in R1 and R2, V22 and V21 in the receiving coil can be extracted according to Formulas (5) and (6). V22 is $H_{S}$ in Formula (1), V21 is $H_{P}$ in Formula (1), and then the normalized quadratic field (*PPM*) can be calculated according to Formula (1).

### 3.2. Primary Field Calibration

**Figure 5:**
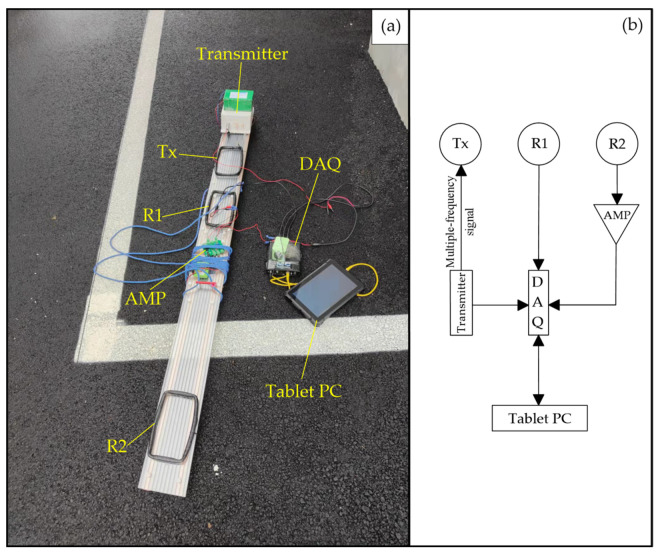
Portable FEM prototype based on digital signal compensation. (**a**) The composition of the prototype; (**b**) signal sending and receiving of the prototype.

According to the device design for digital signal compensation, the prototype shown in Figure 5a was made. The dimensions of the transmitting coil, reference coil, and receiving coil are 20 cm × 10 cm. The seven-frequency pseudo-random signal is transmitted via the transmitter, and the synchronous transmission frequency includes 64 Hz, 128 Hz, 256 Hz, 512 Hz, 1024 Hz, 2048 Hz, and 4096 Hz. As shown in Figure 5b, a data acquisition card (DAQ) is used to directly read the induced electromotive force of the reference coil R1, and the induced electromotive force of the receiving coil R2 is amplified 106 times and then read with the data acquisition card. The data acquisition tasks of the two sets of signals are carried out synchronously.

In order to be able to extract information using Formulas (5) and (6), the primary field in the reference coil and the receiving coil need to be calibrated. The calibration work is to obtain the *n* value in Formula (4). Two questions need to be discussed for *n* values: ① Are *n* values the same in multi-frequency simultaneous operation? ② Should the *n* value contain phase information?

The prototype is mounted on the bottom of a DJI T20 multi-rotor UAV. When the flight height is 100 m, it can be considered that there is no secondary field caused by the geological body in the received signal. Although the number of turns, material, and size of the designed reference coil and the receiving coil are the same, the winding mode of the coil, the mutual inductance between the reference coil and the transmitting coil, the position of the metal material on the prototype, and the distance between the aircraft and the sensor cause differences in the responses of the two receiving coils. As shown in Table 1, the signal ratio between the receiving coil and the reference coil decreases with the increase in the operating frequency, so the *n* value of multi-frequency operation is different. In order to improve the acquisition accuracy of the primary field, the reference coil is close to the transmitting coil, and the mutual inductance between the transmitting coil and the reference coil causes the phase change. Therefore, the *n* value should contain phase information, that is, the *n* value should be complex.

The calibration coefficient and error statistics are shown in Table 2 after 128 superposition calculations with the observation data of 10 s in the air. The relative error of 64 Hz is significantly higher than that of other frequencies, and the main reason for this phenomenon is that the motor rotation of the multi-rotor UAV is unstable. According to the data provided by the DJI, the speed of this motor is generally tens of Hertz, even in the hovering state, and when there is wind, the motor speed of each rotor may change. Therefore, the electromagnetic interference of the multi-rotor UAV is unstable. In order to obtain better-quality calibration data, the detection equipment should be kept away from the multi-rotor UAV, as far as possible, and the calibration operation time should be extended.

After the calibration of the primary field, the *n* in Formula (4) can be obtained. According to the actual measured EMF in R1 and R2, the $H_{S}$ and $H_{P}$ can be calculated using Formulas (5) and (6), and the *PPM* can be calculated using Formula (1).

## 4. Sounding Depth Analysis

Most scholars discuss the detection depth of portable frequency domain electromagnetic methods based on the skin depth of the electromagnetic wave propagation. Huang believes that the sounding depth of a portable electromagnetic method can be approximated as [19]

(7) $$h=\alpha \delta^{\beta }\approx \;\sqrt{\delta }$$

where $\delta =\sqrt{2/\sigma \mu \omega }\approx 503\sqrt{\rho_{S}/f}$, α=0.94, β=0.53, and $\rho_{S}$ is the apparent resistivity. When studying the sounding depth with an airborne electromagnetic method in the frequency domain, Sengpiel proposed the concepts of false layer conductivity and centroid depth based on the analytic solution response of horizontal coplanar one-dimensional layered media, and the centroid depth was also calculated based on the skin depth [25]. The above research and discussion on the sounding depth only consider the frequency characteristics of electromagnetic wave propagation, ignoring the problem of transmitting and receiving capability. When the transmitting current is small or the receiving capacity is weak, the weak secondary field cannot be effectively received. Therefore, it is necessary to discuss the sounding depth based on the transmitting and receiving capability of the device.

**Figure 6:**
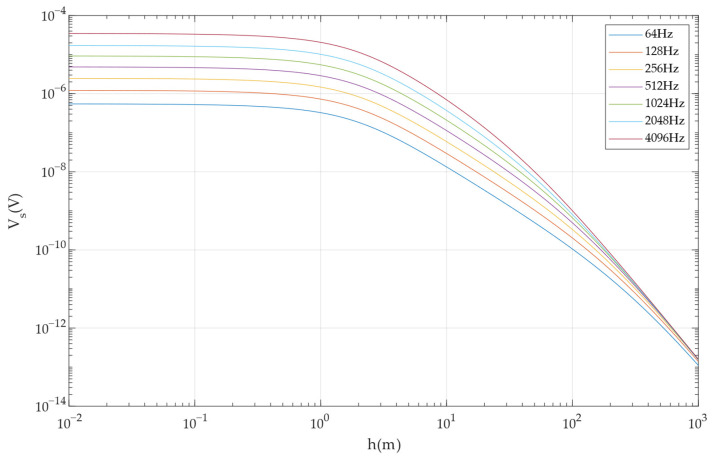
Secondary-field-induced electromotive force of 10 Ω·m geological body at different depths.

Based on the analytical response of the secondary field of the one-dimensional layered medium of the horizontal coplanar device, when the target body is infinitely extended and the resistivity is 10 Ω·m, the influence of the medium between the target body and the detection device is ignored (this layer is considered to be air), and according to the parameters of the detection device made and the emission current of each frequency in Table 1, the secondary-field-induced electromotive force received by the target body at different depths is calculated, as shown in Figure 6.

The secondary-field-induced electromotive force with a uniform half-space resistivity of 100 Ω·m can also be calculated. Using 10 nV as the minimum signal detection capability, the maximum distance for detecting targets with different resistivities is calculated, as shown in Table 3.

The calculated sounding depth based on the skin depth does not take into account the problem of transmitting and receiving capabilities, and the calculated sounding depth gradually increases with the decrease in frequency, but this is contrary to the law in Table 3, wherein the lower the frequency, the closer the detection distance. When the resistivity is 100 Ω·m, Huang’s empirical Formula (10) is used to calculate the sounding depth of 64 Hz as 25 m, which is obviously different from the value of 0.16 m in Table 3. In order to explain the reason why the detection depth decreases with the decrease in the operating frequency, one-dimensional forward modeling is used to calculate the minimum emission current required to detect targets with different resistivities, as shown in Table 4.

As shown in Table 4, detecting the same geological body requires a larger emission current at a low frequency than at a high frequency. If the minimum emission current requirement is not met, the secondary field caused by the target body cannot be effectively received. When the distance from the transmitting coil is further away, the intensity of the primary field received by the geological body is weaker, resulting in a smaller secondary field generated. Therefore, a larger emission current is needed to realize long-distance detection. Compared with the large current required for a low frequency, the small current required for a high frequency is easier to meet, so the lower the frequency, the closer the sounding depth will be. Because the sounding depth is closely related to the device parameters and the emission current, a joint table of the operating frequency, apparent resistivity, and sounding depth can be established by combining the device and emission current parameters, and the sounding depth can be obtained by looking up the table according to the actual frequency and apparent resistivity.

## 5. Tests against Known Targets

### 5.1. Underground Garage Survey

In order to verify the effectiveness of the prototype made based on the digital compensation technology in the actual detection, the detection work was carried out in a known underground garage. The roof of the underground garage was known to be 180 mm thick reinforced concrete. The detection work was centered on the underground garage, and the starting point and end point of the detection were outside the underground garage. The measuring line was approximately straight, and the ground was asphalt pavement. After field measurements, the thickness from the top of the underground garage to the ground was approximately 50 cm, and the steel bar was approximately 20 cm below the ground.

(1) Analog signal compensation

**Figure 7:**
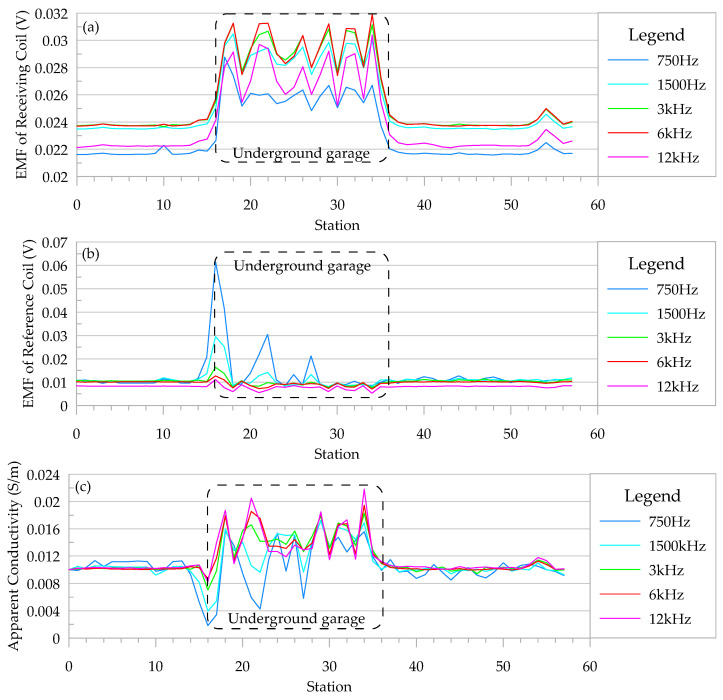
Abnormalities caused by underground parking under simulated signal compensation conditions. (**a**) EMF of receiving coil; (**b**) EMF of reference coil; and (**c**) apparent conductivity.

The walking speed in the detection work was approximately 1.5 m/s, and the height of the detection equipment was approximately 15 cm above the ground. After imitating the analog signal compensation method of GEM-2, the observed EMF of the receiving coil and the EMF of the reference coil as well as the calculated apparent resistivity are shown in Figure 7.

The induced electromotive force (EMF) of the receiving coil was obtained with the two coils in series in reverse. The induced EMF anomaly was found in the upper part of the underground garage. In analog signal compensation, the EMF received in the reference coil should contain only the primary field generated by the transmitting coil and will not change significantly even when the strong eddy current field is encountered. However, as can be seen in Figure 7b, there was an obvious anomaly in the EMF of the reference coil in the actual work.

Because the secondary field in the reference coil is ignored in analog signal compensation, the calculated apparent conductivity is low. As can be seen in Figure 7c, there were obvious abnormalities in the apparent conductivity when passing through the underground garage, and the overall performance displayed an increase in the apparent resistivity, which was a high apparent conductivity anomaly caused by the eddy current field generated by the steel bars on the roof of the underground garage. In this profile, the normal value of the apparent conductivity was approximately 0.01 S/m, and the maximum value of the apparent conductivity generated by the steel bar was 0.022 S/m. The apparent conductivity anomalies caused by the underground garage could be clearly identified, but the intensity of the anomalies was weak.

(2) Digital signal compensation

**Figure 8:**
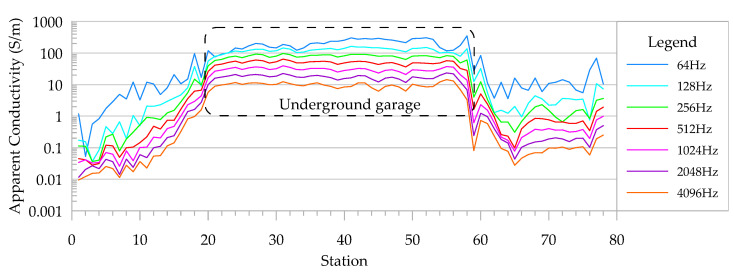
Apparent conductivity curve of underground garage survey.

**Figure 9:**
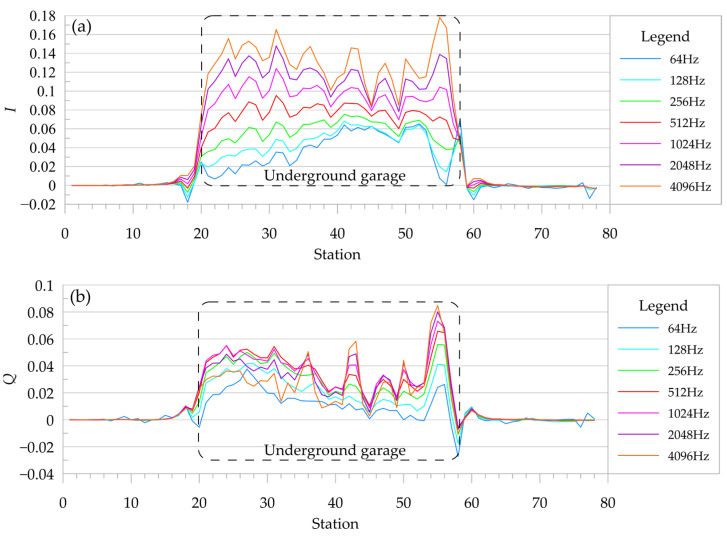
Normalized secondary field curve of underground garage survey. (**a**) The real component of normalized secondary field; (**b**) the virtual component of normalized secondary field.

A re-survey of the underground garage using the digital signal compensation system was carried out. During the detection process, the walking speed was approximately 1 m/s, and the height of the device was approximately 15 cm above the ground. Data were extracted in 1 s, and the station number corresponded to the working time (seconds). The changes in the apparent conductivity and the real and imaginary components of the normalized secondary field caused by the underground garage are shown in Figure 8 and Figure 9.

As shown in Figure 8, the steel bar in the roof of the underground garage caused obvious high apparent conductivity anomalies, and the apparent conductivity gradually increased as the starting end approached the underground garage. The apparent conductivity of 64 Hz was the highest, and the apparent conductivity gradually decreased with the increase in the frequency. The conductivity changes at frequencies above 512 Hz were basically the same. The conductivity stability of the frequency below 512 Hz was poor, and the apparent conductivity above the steel bar was relatively stable. The conductivity of the steel bar was high, generally greater than 10 S/m. Compared with analog signal compensation, the apparent conductivity caused by digital signal compensation was higher, and obvious field value changes could also occur near the garage, indicating that the apparent conductivity data obtained via digital signal compensation were more reasonable.

As shown in Figure 9, both the real and imaginary components of the normalized secondary field showed obvious high-value anomalies above the underground garage, which were more clear than the apparent conductivity.

In this test, the steel bar in the roof of the underground garage caused abnormally high apparent conductivity, which accords with the physical characteristics of the high conductivity of the steel bar. Compared with analog signal compensation, the apparent conductivity obtained via digital signal compensation was higher and the anomaly was more obvious, which proves that digital signal compensation has better sensitivity.

### 5.2. Identifying Distant Car

**Figure 10:**
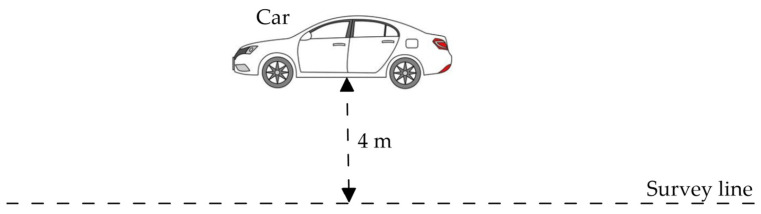
The position between car and survey line.

In order to verify the relationship between the sounding depth and the operating frequency, the test work was carried out on the survey line approximately 4 m away from the car. The device was upright and facing the direction of the car. The position between the survey line and the car is shown in Figure 10.

**Figure 11:**
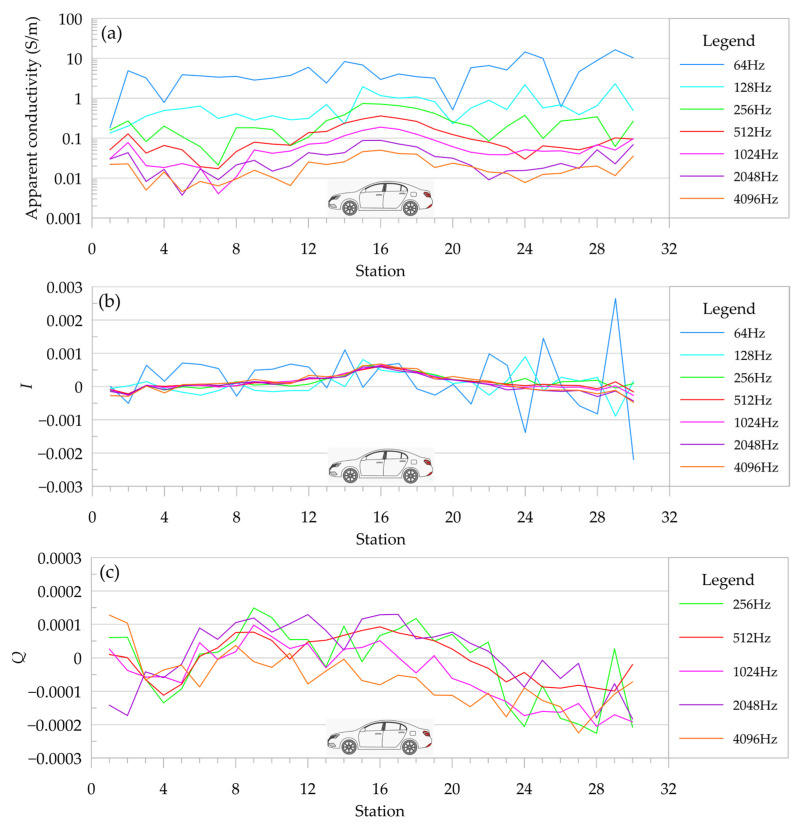
Apparent resistivity and normalized secondary field curves of car survey (the center of the car at station 16). (**a**) Apparent resistivity; (**b**) the real component of normalized secondary field; and (**c**) the virtual component of normalized secondary field.

The survey line was far away from the car, so the anomaly caused was weak. As shown in Figure 11, the amplitude of the apparent conductivity and the real component (I) and the virtual component (Q) of the normalized secondary field changed minimally. Station no. 16 was the center of the car, where the apparent conductivity and the real part of the normalized secondary field had high anomalies. The anomalies in the real component of the normalized secondary field were clear, while the noise in the virtual component was heavy. It can be seen in Figure 11b that the 64 Hz and 128 Hz curves in the real component of the normalized secondary field no longer have an anomaly. The virtual component of the normalized secondary field makes it difficult to identify anomalies caused by the car even after removing the noisy 64 Hz and 128 Hz curves.

The sounding depth calculation based on the skin depth ignores the receiving coil’s ability to receive the secondary field, and it is considered that the lower the frequency, the greater the sounding depth. According to the detection depth Formula (7) proposed by Huang, assuming $\rho_{S}=0.1\;\Omega \cdot m$, the sounding depth at 64 Hz is 4.5 m, and the sounding depth at 512 Hz is 2.7 m. Obviously, the calculated results are contradictory to the experimental results.

In this test, when detecting the weak signal caused by the remote abnormal body, the anomaly could not be distinguished at the low frequencies of 64 Hz and 128 Hz, and the anomaly trend could barely be seen at 256 Hz, while the anomaly at the frequency above 512 Hz was more clear, and this conclusion was consistent with the relationship between the detection distance and the frequency in Table 3. It is proved that it is unreasonable to discuss the depth of detection based only on the skin depth. In the actual detection work of the portable FEM prototype, the lower the operating frequency, the smaller the sounding depth.

## 6. Conclusions

Different from the previous compensation methods of analog signals, a digital signal compensation technology was proposed in this paper, a magnetic dipole horizontal coplanar detection device was optimized, an information extraction method was proposed, and a multi-rotor UAV was used to complete the calibration of the primary field in the air. Based on the digital signal compensation technology, the prototype was made. In the survey of the known underground garage, there were obvious anomalies in the normalized secondary field and apparent conductivity above the underground garage, which proves that the digital signal compensation technology proposed in this paper is effective in practical applications.

In order to analyze the sounding depth of the portable FEM, this paper took 10 nV as the minimum potential detection capability and analyzed the maximum depth of the prototype to detect targets with different resistivities based on the analytical solution response of the magnetic dipole horizontal coplanar one-dimensional layered medium. It was concluded that the sounding depth of the prototype gradually decreased with the decrease in the operating frequency in the survey. This conclusion was confirmed via detection tests that identified a distant car. Therefore, it is necessary to discuss the sounding depth of a portable FEM based on the signal transmitting and receiving capability of the device.

## Figures and Tables

**Table 1:** The response of the reference coil to the receiving coil.

Frequency (Hz)	R1 (V)	R2 (V)	R2/R1	Tx Current (A)
64	0.075	0.096	1.283	0.644
128	0.162	0.207	1.275	0.420
256	0.276	0.345	1.253	0.107
512	0.355	0.425	1.197	0.238
1024	0.258	0.293	1.135	0.357
2048	0.182	0.200	1.097	0.221
4096	0.091	0.098	1.087	0.017

**Table 2:** Calibration coefficients and error statistics.

Frequency (Hz)	n	$\left|n\right|$	Error (V)	Relative Error
64	1.281 − 0.026i	1.283	±0.052	±4.05%
128	1.274 − 0.047i	1.275	±0.005	±0.41%
256	1.248 − 0.081i	1.251	±0.003	±0.22%
512	1.190 − 0.107i	1.195	±0.014	±1.20%
1024	1.128 − 0.094i	1.131	±0.002	±0.22%
2048	1.095 − 0.073i	1.097	±0.001	±0.06%
4096	1.084 − 0.075i	1.087	±0.001	±0.12%

**Table 3:** The maximum distances to detect geological bodies with different resistivities.

Frequency (Hz)	10 Ω·m (m)	100 Ω·m (m)
64	1.56	0.16
128	2.40	0.56
256	3.44	0.97
512	4.71	1.46
1024	6.15	2.08
2048	7.58	2.87
4096	9.14	4.06

**Table 4:** The minimum emission current required for each frequency at 0.5 m distance from the geological body.

Frequency (Hz)	1 Ω·m (A)	10 Ω·m (A)	100 Ω·m (A)
64	1.66 × 10^−1^	1.66	16.6
128	4.15 × 10^−2^	4.15 × 10^−1^	4.15
256	1.04 × 10^−2^	1.04 × 10^−1^	1.04
512	2.60 × 10^−3^	2.60 × 10^−2^	2.59 × 10^−1^
1024	6.53 × 10^−4^	6.49 × 10^−3^	6.49 × 10^−2^
2048	1.64 × 10^−4^	1.62 × 10^−3^	1.62 × 10^−2^
4096	4.14 × 10^−5^	4.06 × 10^−4^	4.05 × 10^−3^

## Data Availability

The data are contained within this article.
